# Malaria infection at parturition in Abeokuta, Nigeria: Current status and pregnancy outcome

**Published:** 2017-08-01

**Authors:** Ayodele S. Babalola, Olufunmilayo A. Idowu, Sammy O. Sam-Wobo, and Eniola Fabusoro

**Affiliations:** 1 Department of Pure and Applied Zoology, Federal University of Agriculture, Abeokuta, Ogun State, Nigeria; 2 Department of Agricultural Extension and Rural Management, Federal University of Agriculture, Abeokuta, Ogun State, Nigeria

## Abstract

**Background:**

There is dearth of information on perinatally acquired malaria, as well as its burden in Nigeria. We determined the prevalence of pregnancy-associated malaria and its burden among parturients in Abeokuta, Ogun State.

**Materials and methods:**

Blood films from 211 parturients were prepared, stained with 10% Giemsa and examined using microscopy. Relevant demographic information was recorded from study participants. Chi-square tests were used to analyse data using SPSS version 20.0.

**Results:**

Prevalence of maternal peripheral, placenta and cord blood parasitaemia were 40.8%, 19.0% and 5.7% respectively, and these were significantly correlated with age and gravidity. Prevalence of maternal anaemia was 45.0%, and was significantly associated with malaria infection. The occurrence of Low Birth Weight (LBW) was 10%. Maternal, placental and cord infections with malaria were associated with LBW, with the highest percentage of LBW occurring in babies with high placental malaria parasite density. Preterm delivery and stillbirth were significantly associated with placenta and cord malaria.

**Conclusions:**

Impact of malaria on the mother and the newborns, notably anaemia and LBW, solicits the need for promoting use of available malaria prevention during pregnancy. These include LLINs and IPTp.

## 1 Introduction

Malaria affects mostly children under the age of five years and pregnant women in sub-Saharan [1,2]. Pregnant women are especially vulnerable because of their reduced natural immunity and are four times more likely to suffer from complications of malaria than non-pregnant women [3]. The destructive consequences of malaria start before the child is born. Children suffer adverse outcomes related to gestational malaria, placental malaria, and congenital malaria. During pregnancy, the acquired anti-malarial immunity of a woman residing in a malaria-endemic area decreases [4]. The risk of malaria increases threefold during the second and third trimesters and fourfold during the initial two post-partum months [5]. This increased threat of malaria is related to an adjustment in the balance of Th1 and Th2 immune factors [6]. The result of these immune modifications is that pregnant women not only suffer from malaria more frequently but they also get sicker when infected by it. The increased risk of severe malaria has considerable and undesirable consequences for both the mother and the developing child [7]. Many pregnant women experience severe anaemia or even death due to severe malaria [3]. Poor fetal development, low birth weight, pre-term delivery, miscarriage, stillbirth, increased rate of anemia during childhood and increased risk for malaria during the neonatal period have been associated with gestational malaria [2,6,8].

Placental malaria has long been recognised as a complication of malaria in pregnancy in areas of stable transmission, and is particularly frequent and more severe in primigravidae [9]. In clinical practice, maternal peripheral parasitaemia is used to detect malaria during pregnancy. However, it has been shown that while peripheral parasitaemia may remain below the levels of microscopic detection, these parasites can be detected in the placenta [10,11].

Studies have shown that *Plasmodium* parasites can cross the placenta leading to mother to child transmission of malaria in a condition known as congenital malaria [12,13]. During delivery, however, blood vessels may burst, leading to mixing of maternal and foetal blood and transmission of parasites from mothers to children [14].

Congenital malaria has been documented in malaria-endemic areas for many years. Studies in Africa have shown that 7–10% of new-borns have malaria parasites in their placental blood and a major part of the transmission of parasites from the mother to the child occurs well before the time of delivery [15,16]. It has also been estimated that 6% of all infant deaths in malaria-endemic areas are caused by malaria infection that took place during the child’s prenatal life [17].

Considering the fact that the consequences of malaria in pregnancy on both the mother and the foetus are enormous and cannot be overemphasised, the prevalence of congenital malaria remains unknown for many parts of tropical Africa [18], including Nigeria [19]. The dearth of information on this issue of great epidemiological importance formed the background against which this study was conducted, i.e. to determine the prevalence of congenital malaria, as well as its burden in a population of parturients in Abeokuta Ogun State, Nigeria.

## 2 Materials and methods

### 2.1 Study area

The research was carried out in Abeokuta, which is the capital of Ogun state. It is situated on the east bank of the Ogun River near a group of rocky outcrops in a wooded savannah, 77 km north of Lagos by railway. Two hospitals, Oba-Ademola Maternity Hospital and State hospital Sokeni Ijaiye were involved with this study.

### 2.2 Inclusion criteria and sample size

Only pregnant women in labour (parturients) were included in the study. A total of 211 parturients were randomly selected within from the two hospitals a timeframe of 3 months, using a formula according to Yamane [20]:


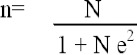


Where,

n = required sample size

N = population size (total number of deliveries in 3 months [477]; obtained from the Secondary Health Management Board, Ijaiye.)

e = the error of 5% points = 0.05

Applying the above formula, the sample size was determined as follows:


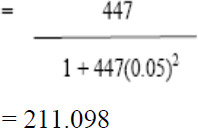


Thus, a sample size of 211 parturients was determined.

The sample population was selected irrespective of age, educational background, marital status, occupation, cultural or religious affiliation.

### 2.3 Procedures

Maternal blood was collected before (for packed cell volume (PCV) determination) and at the onset of labour (for parasitaemia evaluation), into a labelled EDTA bottle, while placental blood was collected using the incision method, after cleaning of the maternal surface with methylated spirit as described by Ezebialu *et al.* [19]. After delivery, cord blood was taken from the umbilical vein (about 15 cm from its place of attachment to the placenta), after cleaning the cord with 70% alcohol to avoid mixing cord blood with maternal blood as described by Theobald *et al.* [18].

Relevant maternal and neonatal demographic and clinical characteristics were recorded in case record forms. Clinical data obtained included details of labour and delivery, anthropometric parameters of the baby and gestational age.

Each baby was weighed naked after delivery using the Baby Weigh TM, electronic scale (MedelaR Inc. model 040.7012). Birth weight below 2500 g was considered to be Low Birth Weight (LBW). Stillbirth was regarded as the birth of an infant that died in the womb (strictly, after having survived through at least the first 28 weeks of pregnancy, earlier instances being regarded as abortion or miscarriage). Preterm birth in this study related to babies born alive before 37 weeks of pregnancy had been completed.

Thick and thin smears were prepared and stained with 10% Giemsa as previously described by Cheesbrough [21]. Blood films were examined microscopically using 100× objectives (with oil immersion). Malaria diagnosis was based on identification of asexual stages of *Plasmodium* species on the thick blood film while thin smears were used for species identification. Slides were declared negative after observing at least 100 high power fields without detecting any parasites. Parasites were counted by estimating the parasite numbers/μl of blood from the thick film as described Greenwood and Armstrong [22].

Packed cell volume (PCV) was determined using blood collected into heparinised capillary tubes and spun with a Hawksley micro-haematocrit centrifuge for 5 minutes. Patients were classified as anaemic when the haemoglobin concentration was less than 11g/dl (PCV<33%), while patients with haemoglobin concentration of less than 7g/dl (PCV<21%) were considered to have severe anaemia.

### 2.4 Data analysis

Data were entered into Excel and thereafter transferred to SPSS version 20.0 for further analysis. Chi-square and binomial regression were used to analyse data. A probability value of P<0.05 was considered significant.

### 2.5 Ethical clearance

Ethical clearance for the study was obtained from the Research and Ethics Committee of the State Hospital Sokenu Ijaiye. All medical personnel of the selected hospitals and study participants were fully briefed on the objectives of the study. Only parturients who gave their written consent were enrolled in the study. Consent forms were given to willing participants after giving them detailed explanation of the research. Consent was obtained in the early phase of labour when pains were least intense.

## 3 Results

The majority of the parturients were within the age range 28 -32 yrs (44.5%), followed by the 23-27 yrs cohort (25.1%). The 18-22 yrs group had the lowest frequency (10.4%). Most parturients were multigravidae (41.2%), followed by primigravidae (31.8%) and secundigravidae (27.0%) (Table 1). All of the parturients were given a Long-Lasting Insecticidal Net (LLIN) during pregnancy but only 59.7% of them used it. 77.7% Of the parturients received IPTp at least once during pregnancy, and the majority (71.3%) only received a single dose of IPTp-SP during pregnancy (Table 1).

**Table 1:** Characteristics of the parturients (n=211) included in the study.

Variable	n	%
*Age (yrs)*		
18-22	22	10.4
23-27	53	25.1
28-32	94	44.5
≥33	42	19.9
*Gravidity*		
Primigravid	67	31.8
Secundigravid	57	27.0
Multigravid	87	41.2
*Use LLIN?*		
Yes	126	59.7
No	85	40.3
IPTp/SP* Use?		
Yes	164	77.7
No	47	22.3
*Frequency of IPTp?*		
Once	117	71.3
Twice	41	25.0
Thrice	6	3.7

* SP=Sulphadoxine-pyrimethamine

The prevalence of maternal peripheral blood malaria parasite infection was 86 (40.8%), while that of placenta and cord blood was 40 (19.0%) and 12 (5.7%), respectively. There was no significant relationship between age and maternal peripheral blood infection. The prevalence of placental and cord malaria parasite infections, however, decrease significantly (p<0.05) with age (Table 2). Prevalence of maternal, placenta and cord malaria parasite infections decreased significantly with increased gravidity (P<0.05); the highest prevalence of maternal (56.7%), placenta (28.4%) and cord blood (7.5%) infections were found in the primigravidae (Table 2).

**Table 2:** Prevalence of maternal, placental and cord malaria infections by age and gravidity.

Variable	# Examined (%)	Malaria infection		
		Maternal n (%)	Placental n (%)	Cord n (%)
*Age (yrs)*				
18-22	22 (10.4)	8 (36.4)	7 (31.8)	3 (13.6)
23-27	53 (25.1)	18 (34.0)	11 (20.8)	0 (0.0)
28-32	94 (44.6)	42 (44.7)	18 (19.1)	8 (8.5)
≥33	42 (19.9)	18 (42.9)	04 (9.5)	1 (2.4)
	p	0.294ns	0.046	0.045
*Gravidity*				
Primigravid	67 (31.8)	38 (56.7)	19 (28.4)	5 (7.5)
Secundigravid	57 (27.0)	28 (49.1)	9 (15.8)	3 (5.3)
Multigravid	87 (41.2)	25 (28.7)	12 (13.8)	4 (4.6)
	p	0.012	0.028	0.498ns

ns = not significant

There was a significant positive correlation between malaria parasite infection of the maternal peripheral blood and occurrence of anemia (r= 0.354; Table 3). There was a significant increase in prevalence of anaemia as parasite density increased (p<0.001). The severity of anaemia increased significantly with increase in malaria parasite density (p<0.05). The prevalence of anaemia reduces significantly (p<0.05) with increase in gravidity with the highest proportion of the primigravid women having anaemia (Table 3). Furthermore, a significant reduction in the prevalence of anaemia occurred as age increased (p<0.001), with the highest proportion of the women with anaemia in the youngest age cohort of 18–22 yrs (Table 3).

**Table 3:** Maternal malaria, gravidity, age and prevalence of anaemia.

Variables	# Examined (%)	Anaemia status	
		Anaemic	Not anaemic
*Malaria status*		p<0.001, r=0.354	
Positive	86 (40.8)	61 (70.9)	25 (29.1)
Negative	125 (59.2)	42 (33.6)	83 (66.4)
Total	211 (100.0)	103 (100.0)	108 (100.0)
*Parasite density*		p<0.001	
Low	12 (14.0)	6 (50.0)	6 (50.0)
Moderate	55 (63.9)	34 (61.8)	21 (38.2)
High	19 (22.1)	17 (89.5)	2 (10.5)
Total	86 (100.0)	57 (66.3)	29 (33.7)
*Gravidity*		p<0.001	
Primigravid	67 (31.8)	53 (79.1)	14 (20.9)
Secundigravid	57 (27.0)	26 (45.6)	31 (54.4)
Multigravid	87 (41.2)	24 (27.6)	63 (72.4)
Total	211 (100.0)	103 (100.0)	108 (100.0)
*Age (yrs)*		p=0.001	
18-22	22 (10.4)	20 (90.9)	2 (9.1)
23-27	53 (25.1)	32 (60.4)	21 (39.6)
28-32	94 (44.5)	35 (37.2)	59 (62.8)
≥33	42 (19.9)	16 (38.1)	26 (61.9)
Total	211 (100.0)	103 (100.0)	108 (100.0)

There was a significant relationship between malaria parasite infection of maternal, placenta and cord blood and birth weight. Out of the 21 babies with LBW, 15 (71.4%), 18 (85.7%) and 9 (42.9%) tested positive for maternal, placental and cord malaria infection, respectively (Table 4). Parturients with low placenta malaria parasite density (<500 p/μl) delivered babies with normal weight, all the babies with LBW were born to mothers with moderate to high placenta malaria parasites density, with the highest percentage from parturients with high (>5000 p/μl) placental malaria parasite density.

**Table 4:** Effects of malarial infection of maternal, placental and cord blood on some measures of pregnancy outcome.

Variable	Examined (%)	Malaria infection		
		Maternal n (%)	Placental n (%)	Cord n (%)
*Birth weight*				
Low				
(< 2.5kg)	21 (10.0)	15 (71.4)	18 (85.7)	9 (42.9)
Normal				
(≥ 2.5kg)	190 (90.0)	71 (37.4)	22 (11.6)	3 (1.6)
	p	0.003	<0.001	<0.001
*Delivery status*				
Preterm	4 (1.9)	3 (75.0)	3 (75.0)	2 (50.0)
Term	207 (98.1)	83 (40.1)	37 (17.9)	10 (4.8)
	p	0.159ns	0.04	<0.001
*Stillbirth*				
Yes	3 (1.4)	3 (100.0)	3 (100.0)	1 (33.3)
No	208 (98.6)	83 (39.9)	37 (17.8)	11 (5.3)
	p	0.035	<0.001	0.037

ns = not significant

A high proportion (75.0%) of the preterm babies were born to mothers infected with malaria parasite, although this was not significant (Table 4). However, placenta and cord infection significantly affected delivery status as 3 (75.0%) and 2 (50.0%) of the babies with preterm delivery had placenta and cord malaria infections, respectively (Table 4). Finally, there was a significant relationship between maternal and placental blood malaria parasite infections and stillbirth as all the cases of still births were from mothers whose peripheral as well as placenta blood were positive for malaria (Table 4).

The result obtained from the univariate analysis on the usage of preventive measures and malaria history during pregnancy showed that non-usage of Intermittent Preventive Treatment (IPTp) is a significant risk factor for placenta malaria (OR= 2.6, 95% CL 1.2-5.4), p=0.018); a higher proportion of the parturients (34.0%) who did not use IPTp tested positive to placental malaria parasite infections (Table 5). We also identified non-usage of LLINs as a significant risk factor for placenta malaria (OR= 2.7, 95% CL 1.3-5.5), p= 0.005).

**Table 5:** Effect of preventive measures on malaria at parturition.

Risk factor	# Examined (%)	+ve for placental malaria (%)	OR (95% CL)	p
*IPT use*	207 (100)			0.018
Yes	160 (77.3)	24 (15.0)	Referent	
No	47 (22.3)	16 (34.0)	2.6 (1.2–5.4)	
*LLIN use*				0.005
Yes	126 (59.7)	16 (12.7)	Referent	
No	85 (40.3)	24 (28.2)	2.7 (1.3–5.5)	

## 4 Discussion

Malaria parasite infections were observed to be prevalent among the parturients in Abeokuta, Ogun State, and was detected in 40.8% of the mothers, 19.0% of the placentas and 5.7% of the cord blood. This shows that malaria in pregnancy is still a serious problem in Abeokuta despite all efforts in place to control it. Pregnancy was previously identified to increase the vulnerability to malaria infections in Abeokuta [23].

The prevalence of placenta malaria recorded in this study (19.0%) is similar to that reported by Olugbenga *et al.* [24] from Nigeria and Alphonse *et al.* [25] from Burkina Faso. Many of the infected placentas (15 of 40) were from parturients who tested negative to peripheral blood parasitaemia. This finding corroborates that of Guyatt and Snow [10] and Bako *et al.* [11]. This could be attributed to the preference of *Plasmodium falciparum* for placenta sequestration and many hypotheses, based on a systemic or local failure of the immunological response to malaria, have been proposed to explain the preference of the parasites for replication in the placenta [9].

The low prevalence of malaria in the umbilical cord (5.1%) recorded in this study is in line with the findings of Falade *et al.* [26]. However, all the malaria-infected cords were from infected placentas. Studies have shown that placenta malaria is the major prerequisite for umbilical cord infection [27]. This is an indication that the placenta barrier may sometimes not be effective against trans-placental transmission of malaria [25]. This was further influenced by placenta malaria parasite density as about 83% (10 out of 12) of the infected cords originated from placentas with high parasite densities. This implies that high placenta parasite density may increase the pressure on the syncytiotrophoblast (separating the maternal circulation from the foetal circulation), thereby reducing its efficiency and consequently facilitating trans-placental transmission of malaria.

In this study, anaemia was associated with malaria parasite infection of maternal peripheral blood. A high percentage of women with malaria infections had anaemia compared with the non infected women. Malaria is directly responsible for anaemia through the parasitic destruction of red blood cells (RBCs) as well as auto immune reaction in which non-infected RBCs are also destroyed.

The prevalence (45%) of anaemia recorded in this study implies that anaemia in pregnancy is a lingering health issue among pregnant women in Abeokuta, Ogun State; there is need for measures to drastically reduce its occurrence. Women often become anaemic during pregnancy because the demand for iron and vitamins is increased in response to physiological changes during that time.

The occurrence of low birth weight (LBW) in the study population was 10%, with the highest prevalence occurring among babies associated with infected placentas (85.7%). Studies have clearly shown that placenta malaria infection is the major cause of malaria related LBW [10,28] which are risk factors for neonatal mortality [29]. Interestingly, the prevalence of LBW increased with increased parasite density of placental infections. This could be attributed to direct mechanical blockage and/or pathological lesions caused by placenta parasitisation [28,30], which in turn compromises the transfer of nutrients to the developing foetus.

Although, the rate of preterm delivery was low (1.9%) it is still important to know that this was significantly associated with placenta and cord malaria. Studies have shown that malaria-infected placentas frequently carry antibodies, cytokines, and macrophages, which are indicative of an active immune response, and this immune response may induce early labour [10]. Women that observed the necessary precautions and preventive measures during pregnancy would have been able to reduce placenta parasitisation, consequently minimising the effect of malaria parasite infections during pregnancy and at birth [31]. Stillbirth in this study (1.4%) was significantly associated with maternal, placenta and cord malaria. The impact of placental malaria on perinatal death (stillbirth and early neonatal death) is still under debate, and conflicting results have been reported from studies that investigated the relationship between these factors [28]. Our study provides additional evidence that placental malaria parasite infection may be responsible for stillbirth. This was further influenced by parasite density as all the stillbirths were from parturients with high placental parasite densities. Furthermore, malaria-induced anaemia can severe the supply of oxygen to the foetus, leading to foetal hypoxia which can result in death. Finally, the risk of placenta malaria parasite infections in the current study was three times higher for parturients that did not use IPTp or LLINs during pregnancy. This buttresses the fact that LLIN and IPT are still very much effective against pregnancy-associated malaria [31]. However, the compliance remained poor in terms of number of doses that were taken so that IPTp can be effective. Similarly, use of bednets, whereby close to half the women were not using a net during pregnancy, leaves much room for improvement.

## 5 Conclusions

Malaria at parturition is prevalent in Abeokuta Ogun State and women below the age of 22 yrs and primigravidae are at highest risk. Maternal anaemia and delivery of LBW babies were the major devastating outcomes of malaria in the peripartum period. Other outcome included preterm delivery and stillbirth. The obvious impact of malaria on the mother and the new-born solicits the need for promoting better use of available malaria preventive measure in pregnancy, notably LLINs and IPTp.
